# Molecular Grammar of Intrinsically Disordered Regions: How Sequence and Context Shape Biomolecular Condensation and Disease

**DOI:** 10.3390/biom16101381

**Published:** 2026-09-22

**Authors:** Wei Liu, Sulin Lou

**Affiliations:** 1 College of Life Sciences and Oceanography, Shenzhen University, Shenzhen 518060, China; 2400171020@mails.szu.edu.cn; 2 Key Laboratory for Plant Epigenetics, Engineering Research Center for Marine Algal Biotechnology, Shenzhen 518060, China

**Keywords:** intrinsically disordered regions, liquid–liquid phase separation, molecular grammar, biomolecular condensates, sequence–function relationship

## Abstract

Intrinsically disordered regions (IDRs) are central components of many biomolecular condensates, where their sequence features help shape the weak and multivalent interactions underlying assembly and regulation. Aromatic and charged residues, sequence patterning, repeat motifs, and spacer properties can influence interaction tendencies and, in defined systems, phase behavior, molecular exchange, and material properties. Post-translational modifications can further tune these interactions. These sequence–interaction relationships provide a basis for the idea of an IDR “molecular grammar.” Here, we use this term specifically for experimentally supported relationships between sequence-level features and interaction tendencies, rather than for the broader cellular processes that organize complex assemblies. In cells, these tendencies operate within multicomponent systems involving nucleic acids, binding partners, stoichiometry, local concentration, and active biochemical processes. We examine how sequence-level molecular grammar contributes to biomolecular condensation and how these contributions are integrated into transcriptional regulation, chromatin organization, RNA metabolism, nucleolar organization, and disease-associated assemblies. We also discuss the evidence needed to connect phase behavior and molecular exchange with material properties and function and compare computational approaches that address different sequence- and assembly-related endpoints. Together, the current evidence supports a layered view in which molecular grammar describes sequence-encoded interaction tendencies, whereas cellular organization and function emerge from the broader molecular and biochemical context.

## 1. Introduction

Biomolecular condensates provide cells with a flexible way to organize biochemical reactions by concentrating selected macromolecules without a surrounding lipid membrane. Liquid–liquid phase separation (LLPS) is one mechanism that can generate such compartments, as demonstrated in reconstituted multivalent protein systems and supported by studies of cellular assemblies [1,2]. A central question in the field is how molecular features encoded in proteins contribute to the formation and physical behavior of these assemblies. Importantly, similar cellular structures can arise through different physical mechanisms, including scaffold binding, polymer bridging or compaction, gelation, and active reaction-driven organization. Thus, punctate or rounded morphology and rapid molecular exchange do not, by themselves, establish LLPS [3,4].

Intrinsically disordered regions (IDRs) are particularly important in this context because their flexible sequences can support multiple weak and reversible interactions. Their effects, however, emerge together with folded domains, nucleic acids, binding partners, and the concentrations and activities of interacting components [5,6,7]. Within this broader context, aromatic residues, charged residues, low-complexity motifs, and the composition of intervening segments can influence molecular interactions and phase behavior. Studies on FUS, hnRNPA1, Ddx4, and related IDR systems have shown that changes in such sequence features can alter phase behavior, although the magnitude and even the direction of these effects depend on sequence background and environmental conditions [8,9,10,11]. Here, we use the term “molecular grammar” specifically to describe experimentally supported relationships between IDR sequence features and molecular interaction tendencies under defined conditions. We distinguish this sequence-level molecular grammar from the broader system-level factors that organize cellular assemblies, including nucleic acids, binding partners, stoichiometry, spatial organization, and active biochemical processes.

Connecting sequence features to condensate behavior also requires appropriate physical measurements. In a simple equilibrium system, the saturation concentration, *C*_sat_, denotes the dilute-phase coexistence concentration under specified conditions, whereas a phase diagram describes how coexistence changes with conditions. Fluorescence recovery after photobleaching (FRAP) reports the recovery dynamics of a labeled component but does not directly measure viscosity, surface tension, or viscoelasticity [12]. These distinctions are important when relating sequence perturbations to changes in assembly and material behavior.

Existing reviews have provided complementary perspectives on condensate organization, molecular driving forces, and the relationship between phase separation and network formation [2,6,13], while methodological studies have clarified the evidence needed to distinguish LLPS from alternative mechanisms in cells [3,4]. An important challenge is therefore to connect these levels of description: how do sequence features alter molecular interactions, how do these changes influence assembly and material behavior, and under what conditions can they be related to biological function? The sticker–spacer framework provides a useful starting point, particularly for defined associative-polymer systems. In more complex assemblies, including transcriptional assemblies, chromatin, stress granules, and nucleoli, heterotypic interactions, stoichiometry, spatial constraints, and energy-consuming processes become additional system-level determinants of organization rather than extensions of sequence-level molecular grammar.

In this review, we organize the current evidence around these sequence–interaction–assembly relationships. We first examine the sequence features and regulatory mechanisms that influence interactions in IDR-containing systems. We then consider how these sequence-level interaction tendencies contribute to physiological and disease-associated assemblies, and how their effects are integrated with multicomponent interactions and active cellular processes. Finally, we compare sequence descriptors, condensate-associated protein classifiers, and single-chain ensemble predictors according to the properties they describe or predict and discuss how their outputs can guide experimental design. This perspective is summarized in a practical predict–validate loop (Figure 1).

## 2. Sequence-Level Molecular Grammar of IDRs

### 2.1. Residue Composition and Interaction Chemistry

#### 2.1.1. Aromatic Residues and π–π/Cation–π Interactions

Aromatic residues (Tyr, Phe, and Trp) and Arg represent one of the best-characterized sequence features within IDR molecular grammar. In several prion-like RNA-binding proteins, their valence and patterning influence multivalent association and phase behavior [8,9,14]. These interactions can involve π–π and cation–π contacts, together with other side-chain, backbone, and solvent-mediated interactions [14,15,16]. Systematic sequence perturbations in FUS-family proteins show that Tyr and Arg valence can alter *C*_sat_ and phase behavior, although the magnitude of these effects depends on sequence background and solution conditions [8,9]. Ddx4 substitutions and designed minimal peptides provide additional examples in which aromatic and cationic residues modulate condensation [10,17]. Arg and Lys are not chemically equivalent in these interactions. Differences in side-chain geometry, charge delocalization, and solvation can lead to distinct cation–π interaction preferences despite their shared positive charge [18,19]. Thus, aromatic–cationic interactions can be considered a context-dependent element of IDR molecular grammar rather than a universal rule. Their contribution is best evaluated together with sequence composition, molecular concentration, partner interactions, and solution conditions.

#### 2.1.2. Charged Residues and Electrostatic Interactions

Within IDR molecular grammar, both the amount and arrangement of charged residues can shape chain conformations and intermolecular interactions [20,21,22,23,24]. In composition-matched polyampholytes, segregating positive and negative residues can strengthen electrostatic attraction, although the outcome also depends on the net charge, hydropathy, solvent conditions, and competing interactions. Charge patterning is commonly described by κ and sequence charge decoration (SCD); these descriptors are useful for comparing sequences but do not by themselves demonstrate LLPS. Ddx4 variants provide a clear example: redistributing charged residues while preserving overall composition can alter phase separation under defined conditions [10,22]. Complex coacervation is a related electrostatic mechanism in which oppositely charged macromolecules accumulate together in a dense phase. This differs from self-association of a single polyampholyte, although similar electrostatic principles can contribute to both processes [23,25]. In LAF-1, protein/RNA composition and salt conditions influence droplet dynamics and measured viscosity [26]. Salt effects are not necessarily monotonic because salt can weaken attractions, screen repulsions, and alter hydration. Thus, charge patterning is best viewed as a context-dependent element of IDR molecular grammar, whose consequences should be evaluated separately at the levels of phase behavior, localization, and function [27,28].

#### 2.1.3. Polar Residues and Hydrogen-Bonding Interactions

Polar residues such as Gln, Asn, Ser, and Thr are common in many prion-like low-complexity domains (PLDs) and can participate in side-chain, backbone, and solvent-mediated hydrogen bonding [13,29,30]. These interactions act together with aromatic, electrostatic, and hydrophobic contacts rather than as an independent driving force. Polar residues therefore represent a context-dependent element of IDR molecular grammar, rather than a uniform class of weak spacers. Mutational and modeling studies show that changes in sequence composition can alter phase behavior but do not support a fixed relationship between a particular polar substitution and *C*_sat_ or material properties [31,32]. Phosphorylation adds another regulatory layer by changing the local charge and interaction chemistry; studies of serine-rich proteins and FUS show that it can alter condensation and aggregation [33,34]. In some condensates, structural transitions and longer-lived contacts can increase network connectivity and viscoelasticity [35]. Such changes may accompany maturation or aggregation, but hydrogen-bonding interactions alone do not imply an inevitable transition toward amyloid formation.

#### 2.1.4. Hydrophobicity, Hydration, and Temperature-Dependent Phase Behavior

Hydrophobic residues influence IDR interactions partly through their effects on solvent exposure and hydration, and their contribution to condensation can vary strongly with sequence and environmental conditions [13,36]. In some systems, hydrophobic motifs provide repeated interaction sites that promote intermolecular association. FG-repeat nucleoporins (FG-Nups), for example, use phenylalanine-rich motifs to form dynamic interaction networks that underlie selective permeability. Depending on sequence and experimental conditions, reconstituted FG-repeat systems can form highly connected or hydrogel-like states rather than simple liquid droplets [37,38].

Hydration is also closely linked to temperature-dependent phase behavior. Elastin-like polypeptides provide a well-defined example in which changes in hydration accompany lower critical solution temperature (LCST) behavior [36,39]. In LCST systems, phase separation occurs above the critical temperature, whereas upper critical solution temperature (UCST) systems separate below the critical temperature. Designed disordered polypeptides further show that sequence composition can shift the transition temperature and, in some cases, alter the type of temperature response [39]. These observations illustrate how hydrophobicity and hydration can form part of a sequence-dependent molecular grammar that links amino acid composition to environmental responsiveness.

Experimental perturbations of hydrophobic interactions require careful interpretation. The aliphatic alcohol 1,6-hexanediol (1,6-HD) can disrupt some condensates, but it also affects enzyme activity and chromatin organization [40,41,42]. Its effects therefore provide supporting evidence for interaction sensitivity rather than a specific test for LLPS. Hydrophobic interactions may also contribute to time-dependent changes in condensate organization and material properties. In some systems, these changes accompany aging, reduced molecular mobility, or fibrillization, but these outcomes depend on the underlying interaction network and should be distinguished from one another [12,29,35,43].

#### 2.1.5. Gly/Pro and Spacer-Controlled Chain Flexibility

Gly and Pro illustrate how spacer chemistry can influence IDR behavior without necessarily acting as strong stickers. By altering backbone flexibility, local structure, and the accessibility of interaction sites, these residues can change how frequently and effectively stickers encounter one another [8,9,11]. Gly generally favors conformational flexibility, whereas Pro imposes stronger geometric constraints and can alter local hydrogen-bonding and cis–trans conformational states. Their effects therefore depend not only on abundance but also on where they occur within a sequence. This distinction becomes especially important in proline-rich regions. Pro-rich motifs can serve as binding sites for folded interaction domains, and the proline-rich domain of Tau contributes to condensation in cells [44]. More broadly, experiments with disordered sticker–spacer polypeptides show that spacer chemistry can tune condensate viscoelasticity [45]. Gly-rich systems can also display time-dependent phase behavior and aging, as shown in a specialized adhesive protein [46]. Together, these studies place Gly and Pro within IDR molecular grammar primarily as modulators of chain properties and interaction geometry, while showing that their roles can shift with the sequence context.

### 2.2. Sequence Patterning, Valence, and Interaction Motifs

#### 2.2.1. Aromatic Valence and Patterning

Aromatic residues often act as stickers in prion-like low-complexity domains (PLDs), and both their valence and their arrangement along the sequence can shape phase behavior [8,9,47,48]. Studies of FUS-related PLDs show that increasing the aromatic valence can strengthen intermolecular association, whereas redistributing the same number of aromatic residues can shift phase behavior by changing how interaction sites are presented along the chain [8,9]. These effects are not determined by aromatic content alone. Residue identity and local sequence context also matter, and a small number of aromatic substitutions can markedly alter condensation in proteins such as TDP-43 [47,48]. In multicomponent mixtures, the outcome depends further on the balance between homotypic and heterotypic interactions, which can promote mixing, selective partitioning, or demixing [49]. Aromatic valence and patterning therefore form a useful part of IDR molecular grammar, linking sequence organization to assembly behavior in a system-specific manner.

#### 2.2.2. Low-Complexity Repeat Motifs (LCRs: RG/RGG and P-Rich Repeats)

Low-complexity repeat motifs encode interaction valence and spacing in a compact sequence format. Arg–Gly (RG) and Arg–Gly–Gly (RGG) motifs are common in RNA-binding proteins, where Arg residues support interactions with RNA and aromatic-rich protein segments. Arg methylation can further tune these associations [50,51,52]. In FUS-related and transcriptional systems, their behavior is also shaped by folded RNA-binding domains, aromatic-rich low-complexity regions, and the C-terminal domain (CTD) of RNA polymerase II (Pol II) [5,53,54]. Proline-rich motifs (PRMs), often embedded in proline-rich domains (PRDs), form a different type of interaction module. Rather than acting through aromatic-like sticker chemistry, PRMs bind structured partners such as Src homology 3 (SH3) domains. Repeated SH3–PRM contacts can drive phase separation in reconstituted multivalent systems and can also promote membrane-associated clustering [1,55]. Thus, repeat motifs contribute to IDR molecular grammar by organizing interaction sites in number and space, while their effects on assembly depend on partner availability, stoichiometry, and the surrounding interaction network.

#### 2.2.3. Multivalency, Phase Separation, and Percolation

Sequence features rarely act in isolation. Their combined effects on assembly depend on interaction valence, partner specificity, linker properties, and stoichiometry. Two related outcomes are phase separation and percolation. Phase separation describes the coexistence of phases with different compositions, whereas percolation describes the formation of a system-spanning network of connected molecules. In multivalent systems, these processes can occur together, but they are not equivalent [6,56]. The sticker–spacer framework and models such as LASSI (lattice model for simulating phase transitions of multivalent proteins) provide a useful way to examine how valence, interaction strength, and linker properties shape these collective behaviors [57]. In multicomponent systems, however, assembly also depends on interactions between different molecular species. RNA, folded domains, and small molecules can bridge components, compete for binding sites, or alter effective valence, thereby promoting or suppressing assembly [5,49,58]. Thus, sequence-level molecular grammar can help define the interaction tendencies of individual components, whereas the collective behavior of multicomponent systems emerges from the broader interaction network.

### 2.3. Regulation of Sequence-Encoded Interactions

#### 2.3.1. Post-Translational Modifications

Post-translational modifications (PTMs) provide a rapid and reversible means of regulating sequence-encoded interaction tendencies. Rather than changing the amino acid sequence itself, PTMs alter the local charge, steric properties, hydrogen-bonding potential, and partner recognition, thereby changing the effective strength and accessibility of interaction sites. In FUS, Arg methylation modifies interactions involving RGG regions and, together with nuclear import receptors, regulates condensation [51,52]. Phosphorylation of the C-terminal domain (CTD) of RNA polymerase II (Pol II) similarly changes its interaction preferences and partitioning during the transition from transcription initiation to elongation and RNA processing [53,59,60]. TDP-43 provides another example of site-specific regulation. Acetylation at Lys136 disrupts RNA binding and promotes pathological assembly, whereas phosphorylation at disease-linked sites can suppress condensation and aggregation [61,62]. At DNA damage sites, poly(ADP-ribose) (PAR) generated by poly(ADP-ribose) polymerase (PARP) enzymes can create multivalent interaction platforms that recruit repair proteins and promote local condensate formation [63,64]. Together, these examples show that PTMs can reversibly modify sequence-encoded interaction tendencies without altering the underlying amino acid sequence. Their effects therefore depend on the modified residue, modification level, and molecular context rather than on the modification type alone.

#### 2.3.2. Alternative Splicing

Alternative splicing can alter the sequence-encoded interaction potential by including or excluding disordered segments, binding motifs, and regulatory sites, thereby generating protein isoforms with distinct interaction landscapes [65,66,67,68]. These isoforms can differ in partner recognition, localization, nucleic-acid binding, condensation, and aggregation. hnRNPDL provides a clear example. Its naturally occurring isoforms show distinct assembly behavior, while structural analysis of hnRNPDL-2 reveals an ordered functional amyloid architecture, illustrating that splicing can redirect an IDR toward different assembly states rather than simply altering the condensation propensity [69,70]. Regulated inclusion of large exons in vertebrate transcription factors provides another example in which changes in extended disordered regions are associated with differences in phase-separation behavior during development [71]. Alternative splicing therefore changes which interaction elements are encoded in an isoform and how they are arranged, while the resulting behavior is further shaped by the cellular environment.

### 2.4. From Sequence-Encoded Interactions to Multicomponent Networks

Cellular condensates are assembled through multicomponent networks in which proteins, RNA, and regulatory ligands continuously modify one another’s interaction opportunities [2,7,72]. In stress granules, G3BP1 (G3BP stress granule assembly factor 1), Caprin1, USP10 (ubiquitin-specific peptidase 10), and RNA form a competitive interaction network whose composition and RNA availability influence assembly [73,74]. Ubiquitin binding to ubiquilin 2 (UBQLN2) provides a different example, in which a ligand can weaken self-association rather than simply add multivalency [75]. At DNA damage sites, poly(ADP-ribose) (PAR) generated by PARP enzymes creates transient multivalent platforms that recruit repair factors and promote local assembly [63,64]. Ongoing transcription, translation, molecular turnover, and ATP-dependent remodeling further reshape these networks over time [76]. These multicomponent networks are therefore distinct from sequence-level molecular grammar itself. Instead, they provide the broader system-level context in which sequence-encoded interaction tendencies are expressed, strengthened, weakened, or redirected. By bridging components, competing for binding sites, and changing effective valence, partner networks can alter assembly composition, even when the underlying protein sequence remains unchanged.

### 2.5. From Sequence-Level Interaction Tendencies to Phase Behavior, Material Properties, and Function

To relate sequence features to condensate behavior, it is useful to separate three questions: does the system undergo phase separation, what are its dynamic and material properties, and does assembly influence biological function? In reconstituted systems, phase separation is best supported by concentration-dependent coexistence, measurements of dilute- and dense-phase concentrations, reversible responses to changes in temperature, salt, or protein concentration, and controls for aggregation or precipitation [3,4,77]. In cells, similar structures can also arise from scaffold binding, polymer compaction, or active processes. Measurements at endogenous or calibrated expression levels, concentration-dependent partitioning, and perturbations that preserve relevant biochemical interactions therefore provide stronger evidence [3,4].

FRAP addresses molecular mobility and exchange rather than material state. Recovery can reflect diffusion, binding and unbinding, and exchange with the surroundings, but it does not directly measure viscosity, surface tension, or viscoelasticity [12]. These properties require independent methods, such as microrheology or deformation–relaxation measurements. Functional links also require more than parallel changes in puncta and phenotype. Stronger evidence comes from assembly-selective perturbations combined with controls for abundance, localization, partner binding, and biochemical activity, together with rescue where possible [4,77]. Keeping these questions separate helps clarify how sequence-level interaction tendencies may contribute to physical behavior and biological function without treating one type of observation as proof of another.

## 3. From Sequence-Level Molecular Grammar to Complex Cellular Organization

The following sections examine how sequence-level molecular grammar contributes to physiological assemblies involved in transcription, chromatin organization, RNA metabolism, nucleolar organization, synaptic signaling, and plant miRNA processing. In these complex cellular systems, sequence-encoded interaction tendencies represent one level of organization and operate alongside binding partners, nucleic acids, stoichiometry, local concentration, spatial constraints, and active cellular processes, which are treated here as system-level factors rather than components of molecular grammar itself. Table 1 summarizes representative examples and the evidence linking sequence-level features, cellular organization, and biological function.

### 3.1. Transcriptional Assemblies and Super-Enhancers

Transcriptional assemblies at super-enhancers provide an example in which sequence-level interaction features contribute to, but do not by themselves determine, cellular organization. Multivalent interactions among transcription factors, coactivators, RNA polymerase II (Pol II), and chromatin contribute to their organization. Phase separation has been proposed as one mechanism in this process [78], while studies of Mediator complex subunit 1 (MED1)-containing coactivator assemblies and live-cell Mediator–Pol II clusters provide experimental evidence for dynamic condensation and transcription-dependent organization [79,80]. The C-terminal domain (CTD) of Pol II provides a clear sequence-level example: its repeated YSPTSPS motifs support multivalent interactions, whereas phosphorylation changes CTD association and partitioning during the transcription cycle [53,59,81]. Transcriptional assembly is further shaped by chromatin binding, coactivators, nascent RNA, local concentration, and ongoing transcriptional activity.

### 3.2. Chromatin Organization

Chromatin provides a useful example of the distinction between sequence-level interaction features and higher-order cellular organization. In heterochromatin, heterochromatin protein 1 alpha (HP1α) recognizes histone H3 lysine-9 di- and trimethylation (H3K9me2/3), while its other regions support oligomerization and nucleic acid interactions. These motif- and modification-dependent interactions illustrate how sequence-level recognition features can contribute to chromatin assembly [82,83,84,85]. Histone acetylation provides another example by changing chromatin interactions and the recruitment of bromodomain readers such as BRD4 [86]. However, chromatin organization is also strongly influenced by polymer compaction, bridging, nucleosome arrangement, and local nuclear conditions. Indeed, heterochromatin can adopt compacted states without clear hallmarks of HP1-driven LLPS [87], and experimental factors such as tags or crowding agents can alter HP1α behavior in reconstitution [88]. Thus, chromatin organization reflects the combined effects of sequence- and modification-dependent interactions, polymer compaction, bridging, nucleosome arrangement, and local nuclear conditions.

### 3.3. RNA Metabolism and Stress Granules

Stress granules (SGs) provide an example in which sequence-encoded interaction tendencies operate within an RNA-rich, multicomponent network. RNA-binding proteins (RBPs) such as G3BP1 combine disordered regions, RNA-binding elements, and regulated protein interactions to control assembly, while RNA availability and conformational changes further tune this process [73,74,89]. RNA–RNA interactions and ATP-dependent remodeling further shape SG composition and dynamics alongside sequence-dependent interactions [76,90]. RNA modification provides an additional regulatory input. N6-methyladenosine (m6A) can enhance the association of YTHDF proteins with multivalent RNAs and promote phase separation in defined systems [91], whereas studies of endogenous transcripts report only limited effects of m6A on SG partitioning [92]. These findings place sequence-encoded motifs and regulated binding interactions at one level of SG organization, while SG assembly and RNA fate are further shaped by the broader protein–RNA network and active remodeling processes.

### 3.4. Nucleolar Organization: Coupling Selective Interactions with Ribosome Biogenesis

The nucleolus is a useful example for showing both the value and the limits of sequence-level molecular grammar. Within the nucleolus, specific molecular interactions help organize different subcompartments. In the granular component (GC), nucleophosmin 1 (NPM1) interacts with rRNA and proteins containing Arg-rich motifs, which supports the recruitment and retention of these molecules in the GC [93,94]. In the dense fibrillar component (DFC), fibrillarin (FBL) associates with nascent pre-rRNA, and these interactions contribute to pre-rRNA sorting and DFC organization [95]. These examples show what sequence-level interaction rules can explain in the nucleolus. They help determine which molecules interact and where they preferentially accumulate.

The organization of the nucleolus, however, also follows the progression of ribosome biogenesis. RNA polymerase I continuously produces nascent pre-rRNA near the FC–DFC boundary. The pre-rRNA is then processed and modified in association with the DFC, followed by the recruitment of ribosomal proteins and assembly factors, ATP-dependent remodeling, and progressive pre-ribosome maturation toward later stages associated with the GC [96,97,98,99,100]. This continuous production and processing of rRNA creates a directional flow of material through the nucleolus, while RNA transport and interfacial effects further shape its subcompartments [101,102]. Thus, selective molecular interactions help organize components locally, whereas the progression of ribosome biogenesis gives the nucleolus its larger spatial and directional organization. Phase organization and ribosome biogenesis therefore work together rather than representing separate processes.

### 3.5. Synaptic Assemblies

Synapses provide an example of how sequence-encoded interaction features contribute to multivalent organization in a membrane-associated environment. Presynaptically, the disordered regions of synapsin interact with synaptic vesicles and partner molecules, and reconstituted systems show that these interactions can generate vesicle-rich condensates [103]. Cellular studies further link synapsin condensation to vesicle sequestration and dynamics [104]. At the postsynaptic density (PSD), modular interactions among scaffolds, receptor-associated proteins, and short binding motifs provide another form of multivalent organization. Reconstituted transmembrane AMPA receptor regulatory protein (TARP)–scaffold systems undergo condensation that promotes selective recruitment and influences synaptic transmission [105]. Tau can further alter the organization and dynamics of PSD-like condensates through multivalent interactions with PSD-95 [106]. These examples show that sequence-encoded multivalency and motif–domain recognition contribute to synaptic assembly, while scaffold stoichiometry, membrane anchoring, and partner composition further shape its organization and dynamics.

### 3.6. Plant Condensates in Small-RNA Processing

*Arabidopsis* dicing bodies (D-bodies) provide a plant example in which sequence-encoded interaction features contribute to the organization of a multicomponent RNA-processing system. SERRATE (SE) contains an intrinsically disordered region and forms condensates in reconstituted systems, while genetic and biochemical studies link SE assembly to the recruitment of DICER-LIKE 1 (DCL1), HYPONASTIC LEAVES 1 (HYL1), and primary miRNA transcripts [107]. This assembly is further regulated by partner proteins. The DEAD-box helicases RH6, RH8, and RH12 promote D-body organization, whereas CYCLOPHILIN71 influences SE condensation through prolyl isomerization [108,109]. Environmental and biological inputs also reshape this network. Heat promotes ARGONAUTE1 (AGO1) assembly, viral proteins can disrupt SE condensation, and SAID1/2 form inhibitory assemblies associated with SE [110,111,112]. In the cytoplasm, SUPPRESSOR OF GENE SILENCING 3 (SGS3) condensation contributes to siRNA-body formation and endogenous gene silencing [113]. These studies show that IDR-dependent interactions contribute to plant small-RNA assemblies, while partner proteins, RNA modification, and environmental inputs further shape their organization and function. Their effects, however, should be interpreted at the level measured. Changes in D-body number, FRAP, or RNA-processing output do not by themselves define shifts in *C*_sat_, critical temperature, or viscoelasticity.

## 4. Disease-Associated Changes in Molecular Interactions and Assembly

Disease-associated mutations can directly change the sequence features that shape molecular interactions. Post-translational modifications and changes in cellular interaction networks can further alter how these molecules interact and assemble. The following sections examine how these different changes contribute to disease-associated assemblies, while distinguishing altered assembly from established causes of cellular dysfunction.

### 4.1. Neurodegenerative Diseases

Amyotrophic lateral sclerosis (ALS) and frontotemporal dementia (FTD) provide well-studied examples of how disease-associated sequence changes and regulatory modifications can alter molecular interactions and pathological assembly. Disease-associated FUS mutations can accelerate the conversion of dynamic condensates or reversible hydrogels into less reversible assemblies in defined experimental systems [114,115]. TDP-43 provides a complementary example. Interactions within its C-terminal low-complexity domain, including a transient α-helical region, contribute to self-association, and disease-linked mutations can alter this behavior [116]. Post-translational modifications can further regulate these interaction tendencies. Lys136 acetylation promotes TDP-43 phase separation and aggregation, whereas disease-linked hyperphosphorylation can suppress condensation and aggregation [61,62], showing that different modifications can shift the same interaction network in opposite directions. Proteostasis factors also reshape this landscape. Ubiquitin binding can alter multivalent interactions, and molecular chaperones can limit aberrant transitions in stress-granule-associated proteins [75,117]. C9orf72-associated Arg-rich dipeptide repeats further perturb stress-granule dynamics, translation, and nucleolar organization through interactions with multiple cellular targets [118,119,120]. These examples illustrate two distinct levels of disease-associated change: mutations can directly alter sequence-encoded interaction tendencies, whereas PTMs, proteostasis factors, and other cellular components modify how these tendencies are expressed within the broader interaction network. The resulting pathological consequences must therefore be interpreted together with downstream effects on RNA metabolism, proteostasis, translation, and other cellular processes.

### 4.2. Cancer-Associated Assemblies

Cancer-associated proteins provide several examples of how changes in sequence-dependent interaction features can contribute to abnormal transcriptional and RNA-processing assemblies. The androgen receptor (AR) provides another example in which its disordered activation domain, coactivator interactions, and pharmacological perturbation influence transcriptional assembly [121]. More direct functional evidence comes from nucleoporin 98 (NUP98) fusion proteins. Their multivalent regions promote nuclear assemblies associated with altered gene expression, and disrupting these interactions reduces leukemic transformation in experimental models [122,123]. A-kinase anchoring protein 95 (AKAP95) similarly links sequence-dependent assembly properties with RNA splicing and tumor growth [124]. Together, these studies show that sequence-dependent multivalency and interaction motifs can contribute to oncogenic assembly, while chromatin binding, partner composition, transcription, RNA processing, and protein stability help determine its cellular consequences. The same distinction is important when interpreting therapeutic perturbations. Bromodomain and extra-terminal (BET) inhibitors and other transcription-targeting compounds can alter both biochemical activity and condensate organization [125,126,127], so changes in condensates should be viewed as one part of a broader drug response rather than as evidence for a single therapeutic mechanism.

### 4.3. Viral Infection and Innate Immunity

The SARS-CoV-2 nucleocapsid (N) protein provides a viral example of how sequence-encoded interaction features contribute to RNA–protein assembly. Its charged and disordered regions support multivalent interactions with viral RNA, and both RNA sequence and structure influence condensation in reconstituted systems [128,129,130]. N assembly is further shaped by phosphorylation, RNA stoichiometry, and interactions with other viral proteins. In particular, N can form RNA-rich condensates containing viral polymerase, whereas interaction with the membrane-associated M protein favors a distinct assembly state [131,132]. These observations indicate that N-protein sequence features help define interaction tendencies, while RNA properties, phosphorylation, stoichiometry, and viral partners further shape the resulting assembly state. N has also been linked to innate immune regulation, including mitochondrial antiviral-signaling protein (MAVS)-associated pathways, although not all N-dependent immune effects can be attributed specifically to condensation [133]. The compound gallocatechin gallate (GCG) can disrupt N condensation and reduce viral replication in experimental systems [134], while analyses of natural N variants further show that sequence changes can alter its biophysical behavior [135]. Together, these studies show that viral sequence changes can alter molecular interactions and assembly, whereas their consequences for viral replication and innate immunity depend on the broader viral RNA, protein, and host-response network.

## 5. Sequence Analysis, Prediction, and Experimental Validation

### 5.1. Sequence Analysis and Endpoint-Specific Prediction

Computational analysis is most useful when the biological question is defined before a tool is chosen. Sequence descriptors such as net charge per residue (NCPR), κ, and sequence charge decoration (SCD) summarize different aspects of charge content and patterning; they are not classifiers of phase separation [20,24,136]. NCPR measures overall charge balance, and κ describes charge segregation. SCD accounts for the positions and separation of charged residues. Their relationships with chain dimensions and phase behavior depend on the sequence context, including hydropathy and the balance between intra- and intermolecular interactions [137,138]. These descriptors are therefore most useful for designing controlled sequence variants and testing how charge patterning affects behavior.

Sequence-based prediction tools address different questions. PScore estimates π-contact propensity from structural statistics and includes interactions beyond aromatic residues alone [14]. PLAAC identifies prion-like amino acid composition and was not developed as a direct LLPS predictor [139]. FuzDrop estimates droplet-promoting and aggregation-related tendencies from the sequence, while its AlphaFold implementation provides a structural visualization of these scores rather than a measurement of material state [140,141]. A complementary approach combining π-contact and β-pairing propensities improved classification in the datasets tested and suggested a possible role for conformational entropy in stabilizing heterogeneous droplet states [142]. These methods can help identify candidate regions, but their scores should remain separate because they describe different sequence properties.

Machine-learning models also differ in their predicted endpoints. PSAP performs protein-level classification [143]. PhaSePred distinguishes self-assembling and partner-dependent proteins [144]. LLPhyScore estimates an interaction-based score for IDR-driven phase separation [145]. PSPHunter combines protein classification with residue prioritization [146], and PICNIC predicts cellular condensate membership [147]. In contrast, ALBATROSS predicts ensemble-averaged single-chain properties such as radius of gyration and end-to-end distance, not phase coexistence or condensate material properties [148]. Model performance also depends on training labels, negative-set construction, sequence redundancy, and database overlap [149]. Table 2 summarizes the endpoint addressed by each method, its data and validation basis, typical applications, and key limitations.

A practical predict–validate workflow should begin with the question being asked. Charge descriptors are appropriate for testing sequence patterning. PScore, PLAAC, or FuzDrop can prioritize candidate sequence regions. Protein-level models can screen candidates or residues, and ALBATROSS can estimate single-chain dimensions. Tool version, construct sequence, domain boundaries, species, and experimental context should be recorded. Prediction then guides, rather than replaces, validation. In reconstituted systems, phase behavior should be tested by measuring coexisting dilute- and dense-phase concentrations across relevant conditions such as salt, pH, temperature, RNA concentration, and stoichiometry. FRAP measures molecular recovery and exchange, whereas viscosity, viscoelasticity, and interfacial properties require independent measurements [12]. In cells, endogenous or calibrated expression, concentration dependence, partner binding, localization, and biochemical activity should be considered, with orthogonal perturbation or rescue used when testing functional causality [4,77].

Databases should likewise be matched to their evidence type. LLPSDB records in vitro phase-separation experiments. PhaSePro curates experimentally supported drivers and phase-separating regions. DrLLPS assigns condensate components with different roles and evidence levels. PhaSepDB collects phase-separation-related proteins and annotations, and RPS focuses on RNAs associated with phase separation [150,151,152,153,154]. Database membership is therefore an evidence resource rather than a direct measurement of phase behavior. Used in this way, sequence analysis and prediction can generate testable hypotheses about sequence-encoded interaction tendencies, while experiments determine whether these predictions extend to phase behavior and complex cellular assemblies.

### 5.2. Experimental Validation, Design, and Intervention

Design should begin with a defined endpoint and an experimentally supported sequence–interaction relationship rather than a presumed universal sequence rule. In systems such as FUS and hnRNPA1, aromatic valence, sticker arrangement, spacer chemistry, and charge patterning provide useful variables for sequence engineering [8,9,11,20,27,28]. A practical strategy is to change one variable at a time, using composition-preserving pattern variants or matched substitutions while keeping expression, localization, and partner binding as constant as possible. The intended endpoint should also be specified in advance, such as phase coexistence, selective recruitment, molecular exchange, mechanical response, or biochemical activity. Because these properties are not equivalent, improving one does not necessarily improve another.

At the cellular level, engineered multivalent modules and optogenetic systems allow for controlled changes in valence, concentration, and spatial recruitment. Domain–motif networks demonstrate how multivalency can tune assembly [1], while optoDroplets enable light-dependent induction and mapping of intracellular phase behavior [155]. OptoMBP provides a complementary approach for reversible condensate dissolution [156]. These tools are most informative when expression is calibrated and appropriate light-only, module, reversibility, and native-protein controls are included. The engineered tag, oligomerization module, and imposed local concentration are part of the experimental system, so induced puncta or a functional response should not by themselves be taken as evidence that the endogenous assembly follows the same mechanism.

Pharmacological intervention requires a similar separation of questions: what does the compound bind, does it alter assembly, and is that assembly change required for the functional effect? Small molecules can partition selectively into condensates, making local enrichment relevant to target exposure [58], but partitioning alone does not establish efficacy or whole-organism pharmacokinetics. Evidence for a condensate-mediated mechanism is stronger when target engagement, assembly-sensitive perturbation, and functional rescue are combined with controls for direct effects on binding or catalysis [4,77]. A practical design cycle is therefore to define the endpoint, introduce minimal sequence or interaction changes, measure phase behavior and material properties with appropriate assays, test function in the relevant cellular context, and revise the model from the resulting data. In this way, experimentally supported sequence–interaction relationships can guide iterative design, while their extension to cellular organization, function, or therapeutic intervention requires validation in the relevant system.

## 6. Conclusions and Future Perspectives

The central message of this review is that molecular grammar is most useful when it is used to describe experimentally supported relationships between sequence-level features and molecular interaction tendencies under defined conditions. Aromatic and charged residues, repeat motifs, spacer properties, and sequence patterning can shape these tendencies, while post-translational modifications and alternative splicing can further regulate them. In cells, partner binding, nucleic acids, concentration, stoichiometry, spatial organization, and active biochemical processes determine how these interaction tendencies are expressed within larger molecular assemblies. The physiological and pathological examples discussed here therefore support a layered view. Sequence features shape interaction tendencies, regulatory mechanisms modify them, and system-level context determines how they contribute to cellular organization and function. Liquid–liquid phase separation is one important organizing mechanism within this landscape, alongside clustering, scaffold-mediated recruitment, polymer compaction, gelation, and active nonequilibrium organization. Phase coexistence, molecular exchange, mechanical properties, and biological function should likewise be treated as related but distinct levels of evidence.

Three directions may be especially important for moving the field forward. First, sequence-level relationships should be quantified more precisely, and their predictive value should be tested across increasingly complex systems. Relationships between features such as aromatic valence, charge patterning, and spacer chemistry and measurable interaction or phase behavior have been demonstrated in defined systems, whereas their ability to predict organization and function in endogenous multicomponent assemblies remains system-dependent and requires direct validation. Second, prediction should move from broad “LLPS propensity” scores toward endpoint-specific models trained and tested on quantitative datasets that preserve the construct sequence, composition, experimental conditions, and assay definitions. Third, engineering and therapeutic studies will benefit from minimal, reversible, and orthogonal perturbations that can separate changes in assembly from changes in binding, catalysis, localization, or expression. These priorities suggest a practical cycle: define the biological question, identify the relevant sequence or interaction variable, predict its effect, measure the physical outcome, test function in the appropriate context, and use discrepancies to refine the model. In this framework, molecular grammar provides testable sequence-level hypotheses rather than universal rules for cellular organization. Its predictive value is strongest where sequence–interaction relationships have been quantitatively established and should be evaluated experimentally when extended to more complex cellular systems. With better quantitative data and causal experiments, this perspective can help move condensate biology from descriptive classification toward predictive, system-specific understanding and rational design.

## Figures and Tables

**Figure 1:**
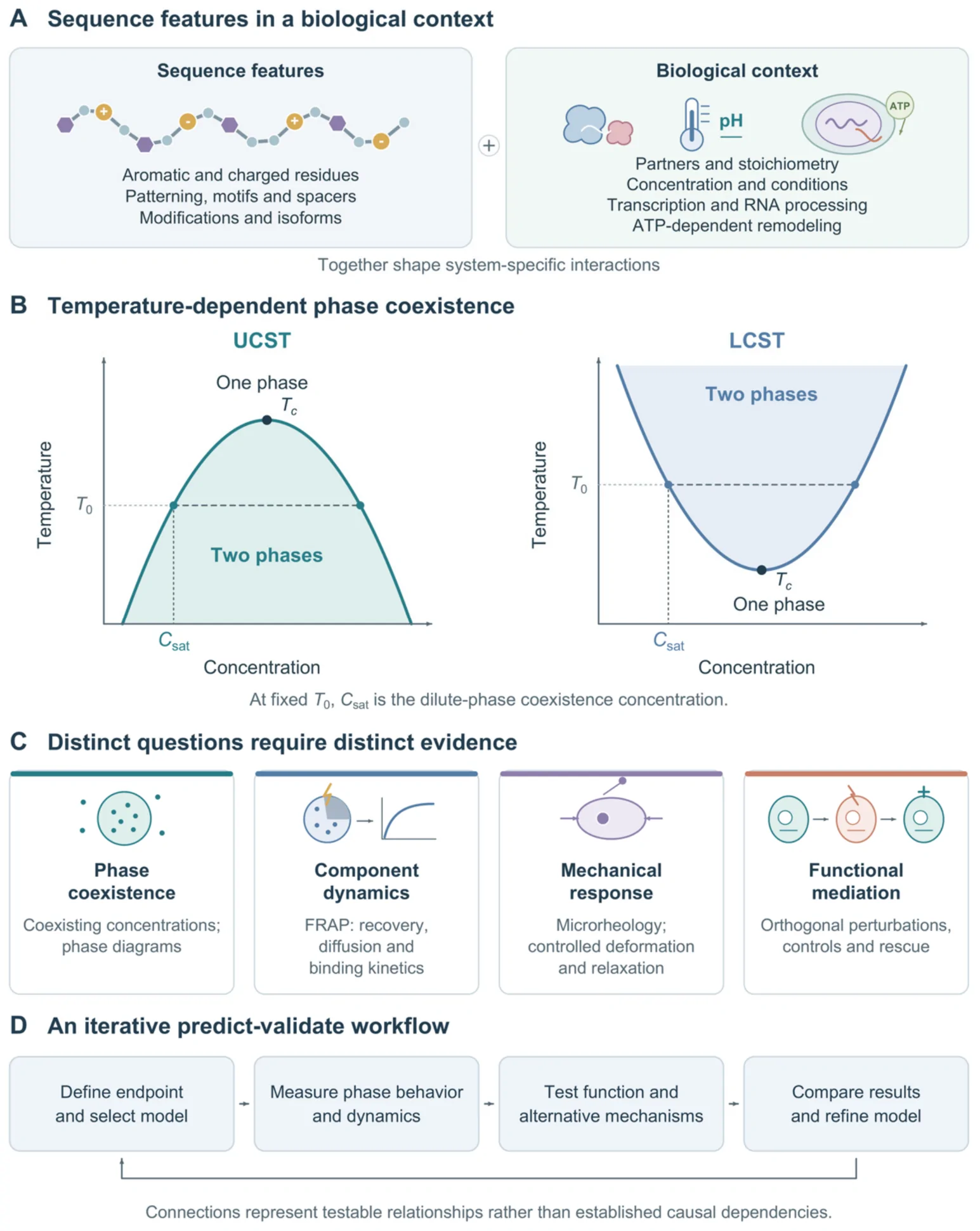
Context-dependent relationships between IDR sequence features, phase behavior, and functional evidence. (**A**) IDR sequence features, including aromatic and charged residues, sequence patterning, motifs, spacers, modifications, and isoforms, contribute to sequence-encoded interaction tendencies. These tendencies operate within a broader biological context that includes binding partners, stoichiometry, concentration, solution conditions, transcription, RNA processing, and ATP-dependent remodeling. In this review, the former are treated as sequence-level molecular grammar, whereas the latter are considered system-level contextual factors. (**B**) Schematic concentration–temperature phase diagrams illustrating upper critical solution temperature (UCST) and lower critical solution temperature (LCST) behavior. Shaded regions indicate two-phase coexistence, whereas unshaded regions indicate a single phase. At a fixed temperature *T*_0_ within the coexistence regime, *C*_sat_ denotes the dilute-phase coexistence concentration. The diagrams are schematic and do not represent experimental data. (**C**) Different properties of biomolecular condensates require distinct types of evidence. Phase coexistence can be assessed from coexisting concentrations and phase diagrams; component dynamics from fluorescence recovery after photobleaching (FRAP), diffusion, and binding kinetics; mechanical response from approaches such as microrheology and controlled deformation or relaxation; and functional mediation from orthogonal perturbations, appropriate controls, and rescue experiments. (**D**) An iterative predict–validate workflow in which a defined endpoint guides model selection, experimental measurements test phase behavior, dynamics, function, and alternative mechanisms, and the resulting data are used to refine the model. Connections represent testable relationships rather than established causal links. Abbreviations: IDR, intrinsically disordered region; UCST, upper critical solution temperature; LCST, lower critical solution temperature; *C*_sat_, saturation concentration; FRAP, fluorescence recovery after photobleaching.

**Table 1:** Representative physiological assemblies: molecular contributors, experimental evidence, and limits of interpretation.

System/Contributors	Sequence-Level Features and System-Level Context	Evidence and Interpretive Limit	Functional Connection
Transcriptional assemblies Pol II CTD, MED1, BRD4	CTD repeats, IDRs, phosphorylation, chromatin and nascent RNA	Reconstitution and cellular clustering/partitioning. Enrichment alone does not distinguish LLPS from binding or active clustering.	Transcriptional recruitment and bursting; separate assembly effects from CTD phosphorylation and coactivator activity.
Heterochromatin HP1 proteins, modified nucleosomes, DNA	Methyl-mark recognition, oligomerization, nucleic-acid binding, polymer topology	LLPS-compatible behavior in some systems; mouse-cell results support alternative compaction states. Tags and crowding matter.	Chromatin organization and silencing; polymer bridging/compaction remain alternative or coupled mechanisms.
Stress granules G3BP proteins, RBPs, untranslated RNAs	RNA availability, regulated protein/RNA contacts, translation and ATP-dependent remodeling	RNA-dependent assembly supported by reconstitution and cells. RNA modification effects are context-dependent.	mRNA partitioning and stress response; translation arrest can precede assembly and cannot be inferred from material state alone.
Nucleolus rDNA, Pol I, pre-rRNA, snoRNPs, ribosomal/assembly proteins; NPM1 and FBL as examples	Selective interactions plus transcription, processing/modification, RNA remodeling and progressive subunit assembly	Coexisting subcompartments and selective interactions; active flux and transcription-dependent organization. NPM1 and FBL are representative contributors rather than a sufficient mechanistic description of nucleolar organization.	Ribosome biogenesis is a hierarchical, energy-dependent pathway; phase organization intersects with biochemical maturation.
Synaptic assemblies Synapsin/vesicles; PSD scaffolds, receptor complexes	Disordered motifs, folded binding modules, membranes and stoichiometry	Defined vesicle or scaffold reconstitutions and cellular studies. Confinement and specific binding contribute.	Vesicle organization and receptor recruitment; synaptic function requires separate tests beyond condensed appearance.
Plant D-bodies SE, DCL1, HYL1, precursor miRNAs and regulators	Protein/RNA interactions, partner activity, modification and stress	SE reconstitution plus genetic/biochemical evidence. D-body number or FRAP does not supply *C*_sat_ or rheology.	miRNA processing; distinguish assembly from altered RNA binding, catalysis, abundance and degradation.

Notes: Components are representative contributors, not a sufficient inventory or a universal set of drivers. *C*_sat_ values are not classified as universally “low” or “intermediate” across unrelated systems. In vitro phase separation, cellular enrichment, molecular exchange, and functional effects are distinguished in the evidence column. Abbreviations are defined in the relevant subsections.

**Table 2:** Distinct endpoints, datasets, validation, and limitations of sequence-analysis methods relevant to condensation.

Method/Endpoint	Data and Validation Basis	Recommended Use and Applicability	Principal Limitation
NCPR, κ, SCD Charge descriptors [20,24,136,137,138]	Mathematical sequence descriptors; assessed using particular polymer models and sequence sets. No universal positive/negative classifier training.	Define composition-matched charge-pattern variants; compare sequences within an appropriate model.	Not direct predictors of LLPS, gelation or rheology. Charge fraction and hydropathy affect interpretation; κ is not domain-spacing.
PScore π-contact propensity [14]	Structural contact statistics from folded-protein structures; evaluated on reported phase-separating proteins and selected controls.	Locate sequence contexts enriched in potential planar-group contacts.	Includes backbone/non-aromatic contacts; not an aromatic-density score, phase diagram, or universal driver classifier.
PLAAC Prion-like composition [139]	Hidden Markov model based on yeast prion-domain composition and background proteome frequencies; validation concerns prion-like domains.	Identify compositionally prion-like segments.	Prion-like composition neither proves LLPS nor predicts a measured material state; background choice affects scores.
FuzDrop Droplet-promoting/aggregation-related regions [140,141]	Sequence-based disorder/binding features; calibrated and evaluated on curated droplet-associated datasets and controls. AlphaFold is used for visualization.	Prioritize candidate regions and hypotheses about competing assembly tendencies.	Scores are not liquid/gel/solid classifications, kinetic trajectories, *C*_sat_ values or direct entropy measurements.
PSAP Protein-level classification [143]	Amino-acid-composition model; 90 literature-curated human positives and the remaining human proteome as background; 10-fold cross-validation plus database and selected experimental comparisons.	Proteome-level candidate screening within a related sequence domain.	Composition/annotation biases and unknown positives in the human proteome background; does not identify a coexistence curve or guarantee cellular driver status.
PhaSePred Self-assembling/partner-dependent classification [144]	PhaSepDB, LLPSDB and PhaSePro; separate SaPS/PdPS training sets (128/214), held-out tests and selected experimental validation. Background lacks PS reports.	Choose the self-assembling or partner-dependent endpoint; document the sequence-only or multimodal model.	Unannotated background can include positives. Some models use non-sequence information; scores are not calibrated *C*_sat_.
LLPhyScore IDR-driven phase-separation score [145]	PDB-derived interaction statistics; 565 curated positive sequences grouped for train/test separation; PDB, human or mixed negatives.	Inspect feature contributions and prioritize IDR perturbations.	Feature contributions are statistical hypotheses, not measured interaction energies. Performance depends on negative-set and sequence-family coverage.
PSPHunter Protein classification + residue prioritization [146]	Curated scaffold sets from four databases/the literature; sequence and functional features; identity filtering and held-out tests; selected in vitro/cellular variant validation.	Select candidate proteins and residues for targeted tests.	Single-domain negatives and annotation-rich features can bias classification. Prioritized residues are not universally proven causal drivers.
PICNIC Cellular condensate membership [147]	CD-CODE positives and protein–protein interaction (PPI) network-selected negatives; sequence/structure features; nonredundant set of 2142 positives/1709 negatives; tests of 24 selected proteins.	Screen potential cellular condensate members, including proteins with little disorder.	Membership includes unresolved driver/client roles. Puncta/localization validation is not proof of autonomous LLPS; selected-sample success is not proteome-wide precision.
ALBATROSS Single-chain ensemble properties [148]	Designed sequences and coarse-grained simulation ensembles; held-out simulations and comparison with experimental chain dimensions.	Estimate radius of gyration, end-to-end distance, asphericity and polymer-scaling exponent.	Simulation/sequence-domain dependence; no prediction of *C*_sat_, intermolecular demixing, viscosity or cellular function.
π-contact + β-pairing model Sequence classification [142]	Compared curated PhaSePro (121 proteins) and merged LLPS (453 proteins) positive sets with selected negatives; cross-validation and dataset comparisons.	Test whether combined structural-propensity features improve discrimination on a defined dataset.	Entropy interpretation is indirect and dataset-dependent; not proof of β structure or a droplet-to-amyloid pathway in a target protein.

Notes: These rows compare published method definitions, not scores on a newly generated common benchmark. Positive and negative labels follow the respective original studies; “negative” usually means unannotated or selected background rather than experimentally unable to condense under every condition. No listed sequence score is a direct measurement of *C*_sat_, viscosity, surface tension, or cellular causality. Database resources are distinguished from predictors in the text.

## Data Availability

Data sharing is not applicable. No new data were created or analyzed in this study.

## Abbreviations

1,6-HD, 1,6-hexanediol; AGO1, ARGONAUTE1; AKAP95, A-kinase anchoring protein 95; ALS, amyotrophic lateral sclerosis; AMPA, α-amino-3-hydroxy-5-methyl-4-isoxazolepropionic acid; AR, androgen receptor; BET, bromodomain and extra-terminal; BRD4, bromodomain-containing protein 4; C9orf72, chromosome 9 open reading frame 72; *C*_sat_, saturation concentration; CTD, C-terminal domain; D-body, dicing body; DCL1, DICER-LIKE 1; DFC, dense fibrillar component; FBL, fibrillarin; FC, fibrillar center; FG-Nup, phenylalanine–glycine repeat nucleoporin; FRAP, fluorescence recovery after photobleaching; FTD, frontotemporal dementia; FUS, fused in sarcoma; GC, granular component; GCG, gallocatechin gallate; G3BP1, G3BP stress granule assembly factor 1; H3K9me2/3, histone H3 lysine-9 di-/trimethylation; HP1α, heterochromatin protein 1 alpha; HYL1, HYPONASTIC LEAVES 1; IDR, intrinsically disordered region; LCR, low-complexity repeat; LCST, lower critical solution temperature; LLPS, liquid–liquid phase separation; LLPSDB, liquid–liquid phase separation database; m6A, N6-methyladenosine; MAVS, mitochondrial antiviral-signaling protein; miRNA, microRNA; NCPR, net charge per residue; NPM1, nucleophosmin 1; NUP98, nucleoporin 98; PAR, poly(ADP-ribose); PARP, poly(ADP-ribose) polymerase; PDB, Protein Data Bank; PLD, prion-like low-complexity domain; PLAAC, prion-like amino acid composition; Pol I/II, RNA polymerase I/II; PPI, protein–protein interaction; PRD, proline-rich domain; PRM, proline-rich motif; PSD, postsynaptic density; PSD-95, postsynaptic density protein 95; PTM, post-translational modification; RBP, RNA-binding protein; RG/RGG, Arg–Gly/Arg–Gly–Gly; rRNA, ribosomal RNA; SARS-CoV-2, severe acute respiratory syndrome coronavirus 2; SCD, sequence charge decoration; SE, SERRATE; SG, stress granule; SGS3, SUPPRESSOR OF GENE SILENCING 3; SH3, Src homology 3; siRNA, small interfering RNA; snoRNP, small nucleolar ribonucleoprotein; *T*_c_, critical temperature; TARP, transmembrane AMPA receptor regulatory protein; TDP-43, TAR DNA-binding protein 43; UBQLN2, ubiquilin 2; UCST, upper critical solution temperature; YTHDF, YTH domain family protein.
